# Utilizing Flaxseed as an Antimicrobial Alternative in Chickens: Integrative Review for *Salmonella enterica* and *Eimeria*

**DOI:** 10.3390/cimb46110732

**Published:** 2024-10-31

**Authors:** William C. Weston, Karen H. Hales, Dale B. Hales

**Affiliations:** 1 Department of Molecular, Cellular & Systemic Physiology, School of Medicine, Southern Illinois University, Carbondale, IL 62901, USA; william.c.weston1@gmail.com; 2 Department of Obstetrics & Gynecology, School of Medicine, Southern Illinois University, Carbondale, IL 62901, USA; karenhales@siu.edu

**Keywords:** infectious disease, poultry immunology, one-carbon metabolism, chicken metabolism, poultry industry, antimicrobial, flaxseed, metformin, salmonella, coccidiosis

## Abstract

This review provides an integrative framework for understanding flaxseed (*Linum utassitissimum*) as an antimicrobial alternative for poultry production. We begin by familiarizing the reader with the global legislation of antibiotics in animal husbandry; highlighting gaps and current issues for *Salmonella enterica* (*S. enterica*) and *Eimeria* (coccidiosis-inducing). We then discuss the natural, symbiotic characteristics of the Galliformes order (chicken-like birds) and *Linum* (the flaxes). The key immunological themes in this review include: (i) flaxseed’s regulation of innate and adaptive immunity in chickens, (ii) flaxseed’s ability to accelerate chicken recovery from infection with *S. enterica* and *Eimeria*, and (iii) flaxseed’s strengthening of immunity via vitamin B6 antagonism. Research indicates that whole flaxseed increases adaptive immune capacity by augmenting cecal Bacteroides and short-chain fatty acids while also attenuating the heterophil to lymphocyte ratio in chickens. Moreover, flaxseed accelerates chicken recovery from infection with *Salmonella* Enteritidis or *Eimeria tenella*; however, future work is needed to better understand (i) defatted flaxseed’s superior performance against *Eimeria* species and (ii) *Eimeria maxima*’s resilience against whole flaxseed. In the context of vitamin B6 antagonism, we propose that 15% whole flaxseed overcomes *S. enterica’s* insult to estrogen synthesis by sustaining the activity of phosphatidylethanolamine methyltransferase (PEMT) in liver. We also propose that 10% defatted flaxseed (as a metformin homologue) strengthens chicken immunity by safeguarding gonadal physiology and by increasing plasma thymidine bioavailability. The concepts in this review can be used as a template for conducting advanced immunological studies in poultry science.

## 1. Introduction

### 1.1. When Scale Greatly Exceeds Carrying Capacity

The global head count of poultry (predominantly broiler chickens) is expected to reach 31 billion head by 2031 (in contrast to 27 billion today), which will provide 47% of all meat protein consumed by humans [1]. Currently, poultry producers slaughter over 70 billion birds per annum in support of human food consumption [2,3]. To understand the magnitude of slaughtering 70 billion birds, consider that the global head count of all wild birds is slightly higher than 50 billion head [4]. The poultry industry’s ecological footprint has motivated researchers to argue that the broiler chicken (as an anthropogenic symbol) represents a blunt reconfiguration of Earth’s biosphere [5]. Despite this, the poultry industry is currently the fastest growing animal market on the planet. From 2023 to 2031, the worldwide consumption of beef, pork, and poultry is expected to rise 9.8%, 11.4%, and 16.7%, respectively [6]. Will this industrial expansion be fueled by the high-intensity production methods of the 20th century (e.g., antibiotic growth promoters (AGPs) [7]), or will agricultural industries seek to harmonize human demand with sustainable alternatives [8,9]?

### 1.2. Antibiotic Usage in Animal Husbandry: A Regulatory Perspective

#### 1.2.1. Progress and Severe Gaps in Legislation

In 1951, shortly after the investigation of AGPs in chickens [7], scientists illustrated that antibiotic overexposure causes drug resistance in turkeys and chickens [10,11]. However, it was not until 1969 that antibiotic resistance in animal husbandry reached federal discussion, when the British parliament distinguished “feed” antibiotics (i.e., AGPs) from medicinal antibiotics [12]. A more poignant concern emerged in the early–mid 1970s, when microbiologists and public health researchers illustrated that humans could develop antimicrobial drug resistance as a result of drug applications in animal husbandry (specifically poultry) [13,14,15].

In 1986, Sweden became the first nation on Earth to prohibit the use of AGPs in animal husbandry, and in 2006, the European Union (EU) adopted similar anti-AGP policies [16,17]. A decade later, in 2017, the United States (US) enacted legislation to prohibit the use of medicinally important antibiotics in animal husbandry, and Japan, South Korea, Canada, Australia, Argentina, Chile, Columbia, and different members of the EU also stratify restrictions by medicinal importance [17,18,19]. China took an important step in 2020 to prohibit the usage of AGPs in animal husbandry; a maneuver that could substantially attenuate the global landscape of AGPs [17,20,21]. However, researchers express concern as to whether China can enforce this new injunction [22].

Lower income nations such as India, Brazil, Uruguay, and the nations of Africa, have little to no regulations for antibiotic usage in animal husbandry [23,24,25]. A key issue in lower income nations is that meat producers commonly perceive their financial prosperity as a direct result of administering antibiotics in animals [23,26,27]. A 2020 systematic review of farms in Africa revealed that antibiotic usage on farms ranged from 78% (for Nigeria) to 100% (for Tanzania, Cameroon, Zambia, Ghana, and Egypt), during the period of 2005 to 2018 [28]. The study also revealed that African nations entirely lack methods to detect emergent drug resistance on farms (i.e., no surveillance). Translating this to public health, a separate 2020 systematic review revealed that pregnant women in Africa experience increased risk of urinary tract infection (UTI) owing frequently to ampicillin-resistant *E. coli* (similar to findings on African farms in [28]) [29].

#### 1.2.2. Is Legislation Using Science Responsibly?

Another issue is a lack of consensus regarding the definition of “antibiotic”. In 2023, the United States Department of Agriculture (USDA) mandated that Tyson Foods (Inc.) stop labeling food products as “no antibiotics ever” when food production had exposed animals to ionophores [30]. This is because the US Food and Drug Administration (USFDA) classifies ionophores as antibiotics. In contrast, the EU classifies ionophores as “anticoccidials”, and therefore, the EU allows businesses to label food products as “no antibiotics ever” when ionophores are used [31]. Why is this noteworthy? Poultry producers often use ionophores as feed additives to inhibit the proliferation of parasites such as *Eimeria* species (coccidiosis-inducing). However, medicated feeds such as these have played a paramount role in the emergence of microbial drug resistance [32,33,34,35].

The USDA’s decision to sanction ionophores in the US food marketplace raises questions. In particular, will US food labeling restrictions (e.g., [30]) act to increase the pharmaceutical sale of *Eimeria* vaccines, or will poultry producers in the US increasingly prefer natural alternatives? These are relevant questions given the common practice of vaccination against *Eimeria* [36,37], as well as the known issues with natural alternatives for *Eimeria* [38,39,40,41]. In contrast, will the EU’s non-sanctioning of ionophores encourage poultry producers to utilize medicated feeds in Europe, or will EU poultry producers gravitate toward natural alternatives regardless? We can gain a clearer understanding of the situation by estimating how legislation and culture differences influence commercial decision making.

#### 1.2.3. Legislation Is Not the End

In November 2023, the Swedish Board of Agriculture ordered Sweden’s largest egg producer, CA Cedergren, to euthanize its entire flock of nearly 1.2 million laying hens (representing 20% of Sweden’s laying hen population). The reason for the mass culling was persistent *Salmonella enterica* subsp. *enterica* (*S. enterica*) serovar contamination [42]. Sweden was the earliest nation to adopt antibiotic-free policies in animal husbandry, suggesting that antibiotic-free poultry production is a well-groomed skill for Swedish producers. Yet, two obvious questions remain. What biological factors were not properly addressed at CA Cedergren, and how did Swedish legislation influence the outcome? One biological factor to consider is that bacteria such as *S. enterica*, *Escherichia coli* (*E. coli*), and *Pseudomonas aeruginosa* (*P. aeruginosa*) pose a persistent threat to poultry production by forming long-lasting biofilms (extracellular matrix communities) on surfaces such as cages, floors, tables, and more. Within these biofilms, bacteria undergo proliferation and genomic recombination (mutation), thus offering an insidious source of infection and drug resistance [43,44,45]. Animals and food products become infected (or contaminated) when they make physical contact with bacterial biofilms. Additional sources of *S. enterica* serovar infection can (but not always) include oral consumption of contaminated feces, mating with infected birds, and the vertical transmission of bacteria from mother to chick [46,47,48,49,50,51]. Indeed, the complexity of bacterial infection is non-linear over time and somewhat unpredictable. This is why legislation cannot be embraced as a final solution. Legislation is designed to support rigid ideals, whereas biological systems tend to be dynamically fluid in relation to context. With this in mind, we offer an integrative review of dietary flaxseed as an antimicrobial alternative in chickens, hoping to inspire a better understanding of flaxseed’s utility as a solution to meet the needs of poultry producers.

## 2. The Galliformes Order (Chicken-like Birds) and Linum (The Flaxes)

### 2.1. Natural Characteristics of Galliformes and Linum: A Symbiosis

One of the earliest ancestors of *Phasianidae* (i.e., pheasant-like birds) was *Asteriornis maastrichtensis*, a chicken-like bird that existed approximately 66.7 to 66.8 million years ago (Ma), prior to the Cretaceous–Paleogene boundary extinction [52]. *Phasianidae* is a subfamily of ground-feeding birds that includes the following genera: *Phasianus* (pheasant), *Gallus* (jungle fowl; chickens), *Meleagris* (turkey), *Parvo* (peafowl), *Coternix* (quail), *Lagopus* (grouse), and *Acryllium* (guinea fowl) [53]. *Phasianidae* is commonly referred to as the avian order “Galliformes”, or also as “galliform birds, gallinaceous birds, and landfowl”. Galliformes, which emerged approximately 67 Ma, stands in contrast with the plant genus *Linum* (the flaxes), which emerged between 27.2 Ma [54] to 46.24 Ma [55]. Therefore, galliform birds underwent geographic dispersion for at least 20 million years prior to *Linum*’s radiation, increasing the likelihood that galliform birds supported *Linum*’s spatial diffusion. *Linum* hosts 180 species of flax that naturally occupy the tropical, subtropical, and temperate climate regions of every habitable continent on Earth. This wide geographic range suggests that avian dieting contributed substantially to *Linum*’s spatial distribution [56,57,58].

Galliform birds are omnivorous ground-foragers that feed regularly on seeds, insects, and insect larvae/pupa [53,59,60]. This opportunist behavior increases the probability of encountering microbial pathogens and environmental toxins. *Linum utassitissimum* (*L. utassitissimum*), also known as “common flax”, hosts a milieu of toxins including linatine, phytic acid, cyanogenic glycoside, protease inhibitor, and cadmium [61,62]. Evidence suggests that flax toxins might be shared similarly across the *Linum* genus [63,64,65]. The outer shell of flaxseed contains a dense mixture of non-starch polysaccharides (insoluble fibers), and this makes the intact seed nutritionally unideal for birds lacking grit in their diet or for small birds in general [66,67,68]. The galliform bird’s large, muscular gizzard and high grit load would make flaxseed an ideal food source [69]. Seed pulverization is essential for accessing the interior dicotyledon fraction where flaxseed’s amino acids and lipids are stored [61,70,71]. Nutrition researchers refer to flaxseed as a “functional food” due to its high density of amino acids and lipids [62,72]. Supporting this, researchers have utilized flaxseed as a 50% replacement for soybean meal in the broiler chick diet (with impressive results) [73].

### 2.2. Parasitic Infection Has Played a Key Role in Chicken Evolution

Since the 19th century, science has observed that domestic chickens (*Gallus gallus domesticus*) are descendants of red junglefowl (*Gallus gallus*), owing to similarities between their appearance, sexual reproduction, and sociality [74]. Validating this, molecular researchers recently genotyped the red junglefowl as the primary ancestor of domestic chickens, although introgression from gray junglefowl, green junglefowl, and Ceylon junglefowl contributed allelic traits during domestication [75,76,77]. The male red junglefowl’s ability to attract female mates is influenced by two key factors: (i) the male’s increased body size and (ii) the male’s comb size and redness [78,79,80,81]. Inoculation of male red junglefowl with the nematode *Ascaridia galli* (*A. galli*) exerts a morphological insult to the male’s comb that greatly influences the female’s mating preference [82]. Specifically, researchers showed that female red junglefowl prefer uninfected males as sexual mates at a ratio of approximately 2:1 (versus infected males), correlating directly with longer, redder combs on males [82]. From an evolutionary viewpoint, the female’s avoidance of infected males helps to prevent (i) female reproductive tract infection, (ii) inflammatory rejection of sperm in the oviduct, and (iii) vertical transfer of infection from mother to chick [51].

By studying *A. galli* infection in industrial laying hens, we can gain further understanding of female red junglefowl mating preference in [82]. Industrial laying hens, when co-infected with *A. galli* and *S.* Enteritidis, exhibit increased fecal shedding of *S.* Enteritidis across time (in contrast to hens that undergo *S.* Enteritidis infection alone) [83]. Therefore, *A. galli* infection multiplies the bird’s shedding of bacteria, and therefore, should act as a multiplier of the infectivity rate. Moreover, *A. galli* establishes itself more severely in laying hens when the animal undergoes co-infection with *S.* Enteritidis [83]. Given these multipliers, we can appreciate why *A. galli* infection acts to modulate the mating preference of female red junglefowl. The *A. galli* protozoal model also has morphological relevance for industrial poultry. For example, infection of poultry with the protozoal genus *Eimeria* causes comb recession, carotenoid loss, and a distinct loss of physical growth [35,41,82,84].

## 3. Flaxseed’s Effect on Innate and Adaptive Immune Function in Chickens

### 3.1. Brief Overview of the Heterophil to Lymphocyte Ratio (H/L) in Chickens: Implications for Gut Microbiome, Short-Chain Fatty Acids (SCFAs), HDACi, and Adaptive Immune Capacity

Heterophils and lymphocytes represent 90% of all leukocytes in vertebrates [85,86,87]. The avian heterophil is referred to as “neutrophil” in mammals. In avian species, the heterophil to lymphocyte ratio (H/L) is a useful biomarker for adaptive immune capacity, stress tolerance, and survivability [88,89,90]. Chicken breeds with an autosomally low H/L display increased resilience against infection from *S.* Enteritidis or *Salmonella* Typhimurium (*S.* Typhimurium) [91,92,93]. Moreover, chicks with a naturally low H/L display increased cecal abundance of Bacteroides, and this abundance is strongly correlated with the cecal concentrations of propionate (r = 0.78) and valerate (r = 0.82) [91]. Propionate (three carbons) and valerate (five carbons) are short-chain fatty acids (SCFAs) derived from the metabolic activity of enteric bacteria. SCFAs regulate adaptive immune function in large part by acting as histone deacetylase inhibitors (HDACis) [94,95]. Nearly all understanding of HDACi modification of immune function is derived from mammalian investigation. However, histone acetylation is conserved in eukaryotes, so we should be able to extrapolate mammalian findings to some extent.

Studies widely suggest that SCFAs and HDACis downregulate the proliferation, differentiation, and function of neutrophils (i.e., heterophils) and myeloid cells, supporting an argument that innate immune capacity undergoes powerful attenuation in response to SCFAs and HDACis [96,97,98,99,100,101]. How do SCFAs and HDACis influence adaptive immunity? SCFAs, via HDACi activity, upregulate the transcription of microRNAs that attenuate B-cell intrinsic function (i.e., antibody-mediated tasks) [95]. Researchers also showed that SCFAs leverage HDAC inhibition to support the proliferation of B-regulatory cells (Bregs) that synthesize interleukin-10 (i.e., B10 cells), thus acting to increase immune tolerance [102]. SCFAs also upregulate B-cell differentiation to Bregs through the activation of free fatty acid 2 (FFA2) receptors, suggesting the involvement of G-protein coupled receptor (GPCR) pathways [94]. These findings support a hypothesis that, in chickens, attenuated H/L is the result of augmented SCFAs acting to (i) inhibit heterophil proliferation and (ii) increase the differentiation of B-cells, culminating in the improvement of adaptive immune capacity.

### 3.2. Whole Flaxseed Strengthens the Adaptive Immune Capacity of Laying Hens by Attenuating the Heterophil to Lymphocyte Ratio (H/L)

Two labs have investigated flaxseed’s effect on H/L, and both investigations were conducted with brown laying hens [103,104]. Researchers in [103] observed that a 3.6% flaxseed diet (g/100 g diet) caused a 28% attenuation of H/L in hens, concomitant with over 60% attenuated TNF-α levels. Even more pronounced, a 5% or 10% flaxseed diet caused a 43% and 36% attenuation of H/L in hens, respectively [104]. Hens consuming either 5% or 10% flaxseed also exhibited an approximately 2.5-fold elevation in antibody titers for sheep red blood cells at 7 days after inoculation. Equally important, the “lymphocyte percentage” (measured as total lymphocytes per total white blood cells) was elevated by 39% when hens consumed 5% flaxseed, bringing into question which lymphocyte compartment(s) was augmented (was it B-cells?) [104]. We suspect that whole flaxseed leverages SCFAs to upregulate B-cell differentiation, according to our observation of a 48% increase in propionyl-carnitine and a 67% increase in valeryl-carnitine in plasma of whole-flaxseed-fed hens [105]. Moreover, our lab used immunohistochemical staining to detect elevated infiltration of CD4+ and CD8+ cytotoxic T-cells within ovarian tumors of flaxseed-fed hens, possibly implicating humoral involvement [106,107]. Future work is needed to determine whether flaxseed increases adaptive immune capacity via B-cell differentiation; however, a good rationale is in place.

## 4. Flaxseed Accelerates Chicken Recovery from Microbial Infection

### 4.1. Flaxseed Versus S. Enteritidis (“Salmonella Infection”)

In 2023, during a 28-day study of *S.* Enteritidis inoculation, researchers used 15% whole flaxseed to accelerate laying hen recovery from *S.* Enteritidis infection by at least 7 days versus control hens (i.e., ≥25% faster recovery) [108]. On day-21 post inoculation (p.i.), *S.* Enteritidis was undetectable in the heart, liver, spleen, and ovary of whole-flaxseed-fed hens, whereas *S.* Enteritidis was detectable in all sampled organs of control hens [108]. By day-28 p.i., *S.* Enteritidis was still detectable in the liver, spleen, ovary, and cecum of control hens, whereas the pathogen was entirely cleared from hens consuming whole flaxseed. Hematoxylin and eosin staining revealed that flaxseed protected the structural integrity of the intestinal mucosal layer, muscularis propria, and outer ileal membrane during infection with *S.* Enteritidis [108]. This protection of ileal barrier function likely helped to minimize the spread of *S.* Enteritidis to the rest of the bird’s organs. Results from [108] also indicate that whole flaxseed increases the cecal abundance of Bacteroides during infection with *S.* Enteritidis, which compliments findings that chickens with a naturally higher cecal abundance of Bacteroides exhibit increased resilience against *S.* Enteritidis [91]. The findings in [91,103,104] suggest that flaxseed’s ability to attenuate H/L also played a protective role in [108]. During future work, it will be helpful to test whether 5% or 10% whole flaxseed are effective against *S.* Enteritidis, and researchers should also test the effects of 5% and 10% defatted flaxseed.

### 4.2. Flaxseed Versus Eimeria (“Coccidiosis Infection”): Strong but Mixed Results

In 1997, researchers observed that 15% whole flaxseed (g/100 g diet) safeguards body weight gain and attenuates intestinal lesions and circulating nitrates in 14-day-old chickens that undergo a 6-day inoculation with *E. tenella* [41]. Similar results were observed during a 13-day inoculation with *E. tenella*, when 10% flaxseed meal and 10% whole flaxseed (g/100 g diet) safeguarded 21-day-old chickens from weight loss, intestinal lesions, and fecal oocysts [109]. In this study, the 10% flaxseed meal diet performed superiorly on all measured outcomes (i.e., lesions, fecal oocysts, and weight gain), including evidence for increased proinflammatory activation (i.e., higher IL-6 and TNF-α upon day-7 p.i.) and efficient inflammatory resolve (i.e., lower IL-1 upon day-7 p.i.) [109]. Moreover, and surprisingly, the 10% flaxseed meal diet caused *E. tenella*-infected chickens to gain more body weight than placebo-infected chickens that consumed the control diet [109]. This is both impressive and paradoxical because pathogen-infected animals typically do not outperform placebo-infected animals. We reviewed the methods of [109] and identified a contributing factor to help explain the high performance of 10% flaxseed meal. Specifically, in Table 1 of [109], the authors leveraged gas chromatography to show that the “mealed flaxseed” (just the seed) contained 2.45% lipids, in contrast to the “whole flaxseed” (just the seed) which contained 28.85% lipids. Therefore, over 91% of the lipids were removed from the mealed flaxseed, which indicates that the flaxseed meal diet from [109] is better understood as a “defatted” flaxseed meal diet. In Section 5.2 and (specifically) Section 5.4, we review the immunological implications of using defatted flaxseed meal as a metformin homologue in the chicken diet. This information could shed light on the superior performance of the 10% flaxseed meal diet in [109].

Although 10% whole flaxseed was effective against *E. tenella* in [109], a separate group of researchers observed that 2%, 5%, and 10% full-fat flaxseed (g/100 g diet) do not protect chickens from *E. tenella* [110]. The authors claimed that 2%, 5%, and 10% full-fat flaxseed lack sufficient omega-3 (n3) polyunsaturated fatty acids (PUFAs) to elicit protection against *E. tenella*. Their claim was supported by work indicating that high exposure to n3 PUFAs protects chickens from *E. tenella*, presumably by promoting oxidative stress in the enteric environment [111,112]. However, the argument for a “minimum threshold of n3 PUFA exposure” is refuted by the findings in [109], where a 10% flaxseed meal diet had 91% of its natural lipids (mainly n3 PUFA) removed, and yet, the diet performed superiorly against *E. tenella*.

It appears that 5%, 10%, or 15% whole flaxseed do not prevent intestinal lesions or prevent weight loss in chickens that undergo *E. maxima* inoculation [41]. Authors claim that *E. maxima*’s colonization of the medial intestine (as opposed to *E. tenella*’s cecal colonization) might challenge whole flaxseed’s usefulness [41]. *Eimeria* species cause unique spatial dysbiosis by colonizing different regions of the intestine. For example, *E. tenella* invades the chicken’s cecum and augments *Campylobacter jejuni* [113], whereas *E. maxima* invades the jejunal (medial) region of the intestine and augments *Heliobacter* species [114,115]. Researchers have not tested flaxseed against *E. acervulina*; however, high levels of dietary n3 PUFA were shown to be ineffective (similar to *E. maxima*) [110]. Chickens can also undergo infection with two or more species of *Eimeria*, leading to unique microbiome alterations and necrotic enteritis [116]. Future research is needed to understand flaxseed’s strengths and weaknesses in relation to spatial dysbiosis. Specifically, researchers need to (i) investigate flaxseed’s utility against *Eimeria* co-infection, (ii) evaluate 10% defatted flaxseed’s utility against *E. maxima*, and (iii) be mindful of the “lipid removal” effects that are encountered when seeds are mealed.

## 5. Understanding Flaxseed’s Anti-Vitamin B6 Effect on Chicken Immunity: A Future Path for Poultry Research

### 5.1. Vitamin B6 Antagonism: A Tool Leveraged by Medicinal Plants and Allopathic Medicine

**Figure 1:**
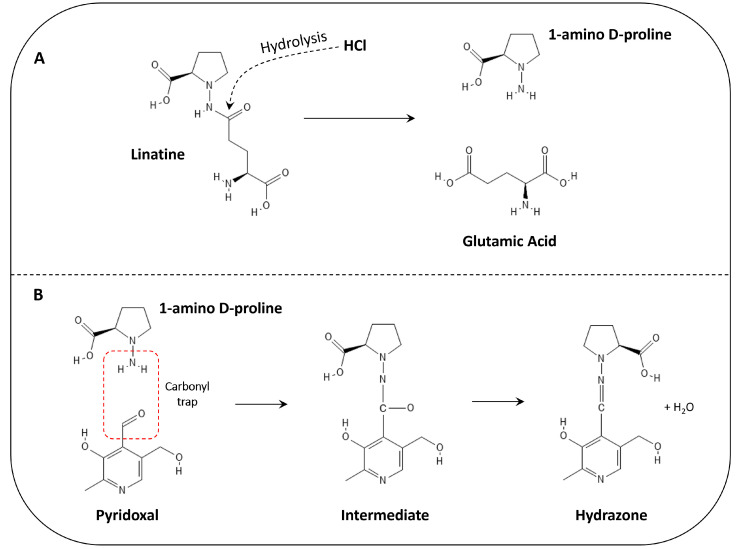
Hydrolysis of linatine (**A**) and the reaction between 1ADP and pyridoxal, leading to hydrazone formation (**B**). This hydrazone can also be formed with PLP.

Flaxseed contains a vitamin B6 antagonizing molecule known as linatine [117]. Linatine is a simple dipeptide consisting of glutamic acid covalently bound to “1-amino D-proline” (1ADP) via a C-N bond (Figure 1). Linatine undergoes rapid hydrolysis in the presence of hydrochloric acid, yielding soluble 1ADP and soluble glutamic acid. In solution, 1ADP can chemically trap the 4′ carbonyl of pyridoxal or pyridoxal 5′-phosphate (PLP), yielding the formation of an inert conjugate (hydrazone) that cannot participate in the vitamin B6 cycle [117,118] (Figure 1). This is the mechanism by which flaxseed attenuates the activity of vitamin B6-dependent enzymes. Another medicinal plant that leverages vitamin B6 antagonism is *Gingko biloba* (*G. biloba*), which contains a powerful vitamin B6 antagonist known as 4′-methylpyridoxine (MPN) [119,120]. Numerous poultry studies indicate that the leaves and leaf extracts of *G. biloba* strengthen humoral immunity, immune tolerance, stress tolerance, intestinal morphology, liver function, and more in chickens [121,122,123,124,125,126,127,128,129,130,131]. The French Lilac (*Galega officinalis*), which contains isoamylene guanidine [132], might also antagonize vitamin B6 based on evidence that metformin (an isoamylene guanidine derivative) blocks the PLP binding site of serine hydroxymethyltransferase 2 (SHMT2) [133]. Moreover, metformin and 1ADP both leverage amino groups to trap carbonyls [117,134,135]. Allopathic human medicine also recognizes vitamin B6 antagonists therapeutically (e.g., cycloserine and isoniazid for tuberculosis; hydralazine for high blood pressure; penicillamine for rheumatoid arthritis; and theophylline for asthma) [136].

### 5.2. Flaxseed’s Anti-Vitamin B6 Effect on One-Carbon Metabolism and Cellular Bioenergetics

In 1986, researchers observed that whole flaxseed causes an extreme elevation of plasma cystathionine in chickens, presumably by antagonizing vitamin B6 [137]. Our lab verified these findings when we observed more than a 15-fold elevation of plasma cystathionine in conjunction with attenuated 4-pyridoxic acid and pyridoxamine when laying hens consumed either 15% whole flaxseed or 10% defatted flaxseed (see Figures 3, 8, and 10 in [138]). Interestingly, the hens did not undergo hyperhomocysteinemia despite a severe accumulation of cystathionine. Instead, the hens compensated by accelerating homocysteine remethylation and S-adenosylmethionine (SAM) synthesis.

Accelerated SAM synthesis can serve as a potent activator of glycolysis and mitochondrial fatty acid oxidation (FAO) in chickens (see Figures 1, 7, and 9 in [105]). Supporting this, multiple studies have shown that accelerated SAM synthesis activates AMP-activated protein kinase (AMPK), presumably in response to ATP deficiency [139,140]. In chickens, AMPK activation upregulates glycolysis and mitochondrial FAO during situations of metabolic stress [141,142]. Moreover, AMPK couples tightly with the immune response to support late-stage inflammatory resolve when chickens are fighting *S.* Enteritidis infection [143]. Recently, we observed evidence that 10% defatted flaxseed functions as a metformin homologue (i.e., AMPK activator) by upregulating glucose uptake and glycolysis in hens [105], while 15% whole flaxseed acts to improve liver function by activating phosphatidylethanolamine methyltransferase (PEMT) in hens [138].

### 5.3. Whole Flaxseed’s Immunological Role as a PEMT Activator: A Case for S. enterica

#### 5.3.1. Review of *S. enterica*’s Insult to the Follicular Granulosa: Implications for Estrogen Synthesis and Hepatic Lipoprotein Metabolism

*S.* Enteritidis and *S.* Typhimurium cause inflammation in tissues via the effects of lipopolysaccharide (LPS) [144,145]. LPS binds to a toll-like receptor (TLR) complex on the surface of myeloid cells, which transduces pro-inflammatory signals that upregulate the secretion of cytokines and chemokines [146,147,148]. Immune infiltrating cells (e.g., heterophils) then surge to the region of inflammatory signaling, which exposes the target tissue to oxidative burst [87,149]. During *S. enterica* infection, oxidative burst within the ovary causes progesterone synthesis to attenuate in the granulosa [150,151,152]. The ovary then undergoes increased F1 follicle death, which compromises the ovary’s ability to generate estrogen [150,151,153,154].

Estrogen is protective against microbial infection according to evidence that hens rapidly clear pre-existing infection upon reaching sexual maturity [155]. Sexual maturity offers the female with increased access to estrogen-responsive genes, in particular, increased access to *PEMT* in the liver [156,157,158,159]. PEMT is a SAM-dependent methyltransferase that consumes three molecules of SAM to tri-methylate one molecule of phosphatidylethanolamine (PE), yielding phosphatidylcholine (PC) [160]. PEMT is cofactor-independent; therefore, PEMT activation is driven by the availability of PE and SAM [161]. Like other vertebrates, the *Gallus gallus PEMT* locus contains an estrogen response element (ERE) palindrome that is proximal to numerous AP-1 enhancers [105,162]. Testing this, 17-beta estradiol was used to upregulate lipogenic genes such as *PEMT* in an ERE-dependent manner in laying hen liver [156]. PEMT is arguably indispensable for stable lipoprotein synthesis. For example, during *PEMT* knockdown/out, apolipoprotein B cannot incorporate into the nascent lipoprotein molecule (owing to PC insufficiency in the lipoprotein membrane), which causes the formation of small, non-viable lipoprotein molecules in the golgi of the hepatocyte [163,164,165,166]. These non-viable lipoprotein molecules struggle to exit the liver, which attenuates the concentration of lipoprotein in circulation [165,166,167,168].

**Figure 2:**
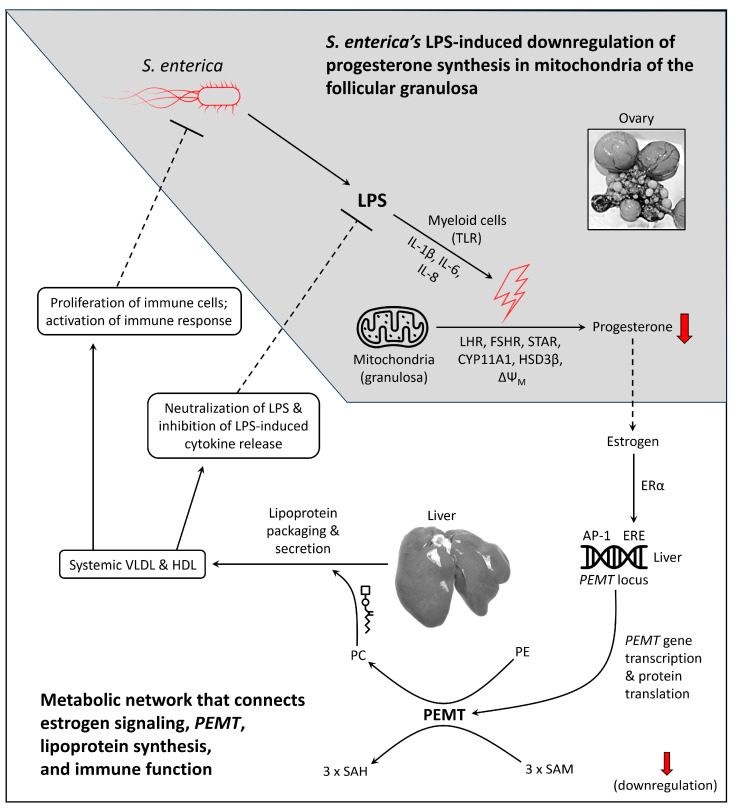
Model of *S. enterica*’s effect on steroidogenesis in the ovarian follicle, contrasted with estrogen’s role in promoting *PEMT* gene transcription, lipoprotein formation, and lipoprotein-mediated inhibition of *S. enterica*. Abbreviations: AP-1 = AP-1 enhancer; CYP11A1 = cytochrome p450 11A1; ERα = estrogen receptor alpha; ERE = estrogen response element; FSHR = follicle-stimulating hormone receptor; HSD3β = 3β-hydroxysteroid dehydrogenase; HDL = high-density lipoprotein; IL-1β = interleukin-1β; IL-8 = interleukin-8; LHR = luteinizing hormone receptor; LPS = lipopolysaccharide; PC = phosphatidylcholine; PE = phosphatidylethanolamine; PEMT = phosphatidylethanolamine methyltransferase; SAH = S-adenosylhomocysteine; SAM = S-adenosylmethionine; TLR = toll-like receptor; ΔΨ_M_ = delta psi (mitochondrial membrane potential). Dashed lines are intended to show change from systemic circulation to the ovarian compartment.

Under normal conditions, liver-secreted very-low-density lipoprotein (VLDL) and high-density lipoprotein (HDL) physically bind to LPS molecules, thereby preventing LPS from activating TLRs [169]. This binding allows lipoprotein to exert a direct anti-inflammatory effect. Another mechanism that connects lipoprotein to immune function is the VLDL-mediated trafficking of cholesterol, phospholipid, and triglyceride. VLDL-trafficked lipids stabilize the fluidity of immune cell membranes, which allows for the appropriate insertion of TLRs, major histocompatibility complex (MHC) class II protein, B-cell receptors, and T-cell receptors [170,171,172]. In this manner, VLDL directly influences the immune cell’s ability to detect and kill pathogens. Additionally, VLDL-derived lipids support proliferative immune states such as when antigen-sensitized CD8+ T-cells undergo clonal expansion [172,173]. In Figure 2, we offer a model that illustrates *S. enterica*’s insult to progesterone synthesis in the follicular granulosa, in contrast to the metabolic network that connects estrogen signaling, *PEMT* gene transcription, lipoprotein formation, and lipoprotein-mediated immune activities.

#### 5.3.2. Whole Flaxseed Accelerates Laying Hen Recovery from *S. enterica* Infection by Sustaining PEMT Activity: A Model for Compensating *S. enterica*’s Insult to Estrogen

**Figure 3:**
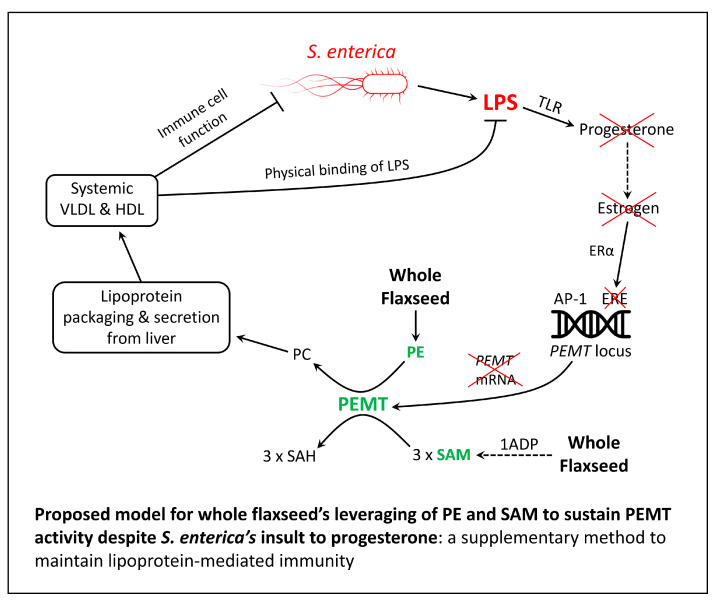
Proposed model of flaxseed’s sustainment of PEMT activity during *S. enterica* infection, culminating in the preservation of lipoprotein synthesis and lipoprotein-mediated immune function. 1ADP = 1-amino D-proline; AP-1 = AP-1 enhancer; ERE = estrogen response element; ERα = estrogen receptor α; HDL = high-density lipoprotein; LPS = lipopolysaccharide; PC = phosphatidylcholine; PE = phosphatidylethanolamine; PEMT = phosphatidylethanolamine methyltransferase; SAH = S-adenosylhomocysteine; SAM = S-adenosylmethionine; VLDL = very-low-density lipoprotein. All instances of a red “X” indicate an inhibitory effect of LPS. The dashed arrow is used to indicate that intermediate steps are required (explained in Figure 8 of [138]).

In Section 5.2, we discussed how flaxseed (via 1ADP) augments one-carbon metabolism to offer increased access to SAM. Moreover, whole flaxseed contains a dense concentration of phospholipid, including PE. Therefore, a 15% whole flaxseed diet is optimal for driving PEMT activity (at a substrate level). This is novel in the context of *S. enterica’s* colonization of the ovary. Subsequent to infection, *S. enterica*’s insult to estrogen would attenuate the transcription of *PEMT* mRNA in hepatocytes, thus indirectly attenuating the synthesis of PC. However, by supplying the liver with extra PE and SAM, whole flaxseed can “hyper-employ” PEMT to compensate for attenuated *PEMT* gene transcription (at least for a given time, likely proportional to PEMT’s protein half-life). This would act to (i) sustain hepatic PC synthesis and lipoprotein formation, (ii) prevent steatosis and liver dysfunction, and (iii) allow VLDL and HDL to conduct the systemic immune tasks described in Section 5.3.1. In Figure 3, we propose a concept whereby whole flaxseed overrides *S. enterica*’s insult to progesterone and estrogen, in particular by continuously supplying PEMT with PE and SAM.

### 5.4. Immune Implications for Defatted Flaxseed as a Metformin Homologue

#### 5.4.1. Review of Metformin’s Immuno-Reproductive Effects in Chickens

In Section 5.2, we reviewed how 10% defatted flaxseed works as a metformin homologue in laying hens. Over the last decade, poultry researchers have endeavored to clarify metformin’s physiological effects in chickens. Most of this work focused on gonadal responses to metformin, which offers reproductive insight into metformin’s regulation of poultry immunity. In non-infected rooster Sertoli cells, a 5 mM dose of metformin was shown to alter the cell’s inflammatory tone by upregulating TLR-1, TLR-2, IL-1β, IL-6, IL-8, and IFN-γ (and notably not TLR-4) [174]. Moreover, metformin can synergize with LPS to additively increase the Sertoli cell’s pro-inflammatory response [174]. Metformin should also improve the chicken’s inflammatory tone by attenuating testosterone (given that testosterone is immuno-suppressive) [175]. For example, roosters exhibit reduced testicular weight and 50% attenuated testosterone after receiving 150 mg/kg metformin for three weeks [176]. Although this seems “gonadally alarming”, a rooster’s fertility might not be entirely compromised by metformin, according to findings that AMPK activators (like metformin) increase rooster sperm motility and acrosomal activity [177,178,179]. Researchers also observed attenuated testosterone in 65-week-old broiler breeder hens that undergo dietary supplementation with 50 and 75 mg/kg metformin [180]. These hens exhibit normalized prehierarchical and preovulatory follicles, coupled with increased progesterone, egg laying, egg hatchability, and reproductive longevity [180].

Poultry scientists used multiple assays (RT-qPCR, Western blot, immuno-precipitation, and transmission electron microscopy) to show that metformin improves reproductive longevity by attenuating atresia in prehierarchical follicles of aged laying hens [181,182]. Similarly, AMPK activators (e.g., nobiletin) have been shown to rescue the expression of genes and proteins that regulate granulosa cell proliferation in prehierarchical follicles of aged hens [183]. Overall, metformin’s ability to safeguard follicle longevity should improve the hen’s daily lifetime exposure to estrogen, and thus offer increased immune protection throughout life [155,184]. More work is needed to study specific immune responses in metformin-treated chickens; however, the current findings indicate a superior platform for immunity.

#### 5.4.2. Defatted Flaxseed as an Accelerator of Immune Cell Expansion: Implications for Increased Thymidine Synthesis

Serine, the cell’s primary source of 5,10-CH_2_THF, plays a critical role in the expansion of effector T-cell populations [185]. In cytoplasm, 5,10-CH_2_THF is the carbon donor that converts deoxyuracil monophosphate (dUMP) to deoxythymidine monophosphate (dTMP), via thymidylate synthase (TS) [186]. TS activity is critical for cell proliferation because thymine (derived from deoxythymidine triphosphate, dTTP) is incorporated into DNA during the S-phase of the cell cycle [187,188]. Inhibition of TS exerts catastrophic effects on peripheral T-cell expansion, which can be rescued with thymidine supplementation [188,189]. During proliferative states, 5,10-CH_2_THF shunts through TS to accelerate dTMP synthesis. When dTMP levels are too high, cells leverage 5′-deoxynucleotidase (5DR) to dephosphorylate dTMP, yielding soluble thymidine and inorganic phosphate [190,191]. When dTMP demand increases, soluble thymidine can be phosphorylated via thymidine kinase, yielding dTMP again [192].

In a human recombinant enzyme system, researchers observed that metformin accelerates several pathways that are involved in thymidine synthesis. Metformin physically blocks the PLP binding site of mitochondrial SHMT (SHMT2), which causes a metabolic shift from SHMT2 to cytosolic SHMT (SHMT1) [133]. This shift to SHMT1 ensures that the cell can still generate 5,10-CH_2_THF; however, it also increases the flux of 5,10-CH_2_THF through methylenetetrahydrofolate reductase (MTHFR), leading to increased synthesis of SAM. When SAM is elevated, it acts as an allosteric inhibitor of MTHFR, which shunts 5,10-CH_2_THF through TS, causing increased synthesis of dTMP [140]. Evidencing this, the researchers in [133] observed that metformin causes simultaneously elevated SAM and dTTP, suggesting increased shunting of 5,10-CH_2_THF through TS due to MTHFR inhibition. This is the manner in which metformin would elevate thymidine synthesis.

**Figure 4:**
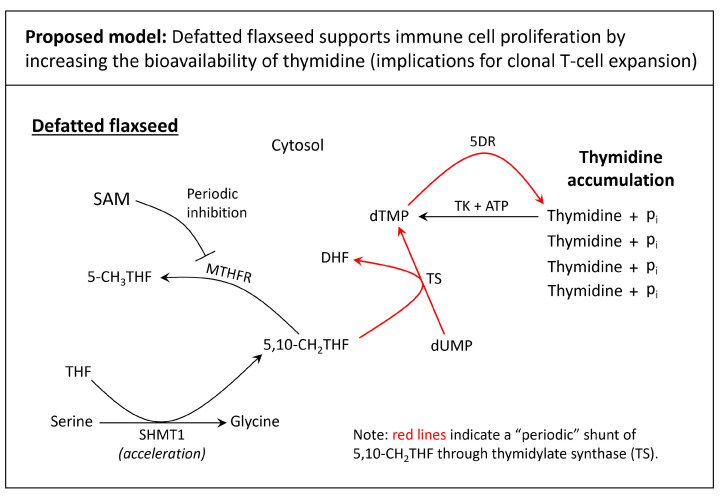
Model for thymidine accumulation when laying hens consume 10% defatted flaxseed. Defatted flaxseed, by accelerating SHMT1, causes the increased synthesis of SAM. Elevated SAM allosterically inhibits MTHFR, which then shunts 5,10-CH_2_THF through TS, yielding increased dTMP synthesis. dTMP can then be converted to thymidine via 5DR. Increased thymidine bioavailability supports the proliferative demands of the immune system subsequent to antigen detection or injury. Abbreviations: 5-CH_3_THF = 5-methyltetrahydrofolate; 5,10-CH_2_THF = 5,10-methylenetetrahydrofolate; 5DR = 5′-deoxyribonuclease; ATP = adenosine triphosphate; DHF = dihydrofolate; dTMP = deoxythymidine monophosphate; dUMP = deoxyuracil monophosphate; MTHFR = methylenetetrahydrofolate reductase; p_i_ = inorganic phosphate; SAM = S-adenosylmethionine; SHMT1 = serine hydroxymethyltransferase 1 (cytosolic SHMT); THF = tetrahydrofolate; TK = thymidine kinase; TS = thymidylate synthase.

Our lab observed moderately elevated plasma thymidine in laying hens that consumed 10% defatted flaxseed. This effect (i.e., thymidine accumulation) was expected because 10% defatted flaxseed also caused a 1.9-fold elevation of SAM in hens [138]. As mentioned above, elevated SAM allosterically inhibits MTHFR, thereby increasing the shunting of 5,10-CH_2_THF through TS, resulting in increased synthesis of dTMP, and thus, increased synthesis of thymidine (via 5DR). Improved thymidine bioavailability supports the proliferation of immune cells, such as when antigen-sensitized T-cells undergo massive clonal expansion [173,187,188]. Our framework for defatted flaxseed (illustrated in Figure 4 below) acts in accordance with the metformin framework in [133]. In light of these similarities, researchers should be able to ask deeper immunological questions about defatted flaxseed’s role as a metformin homologue.

## 6. Conclusions

In this review, we have acquainted the reader with the current state of affairs in antibiotic legislation for animal husbandry, and we sought to illustrate how legislation creates gaps to be filled by antibiotic alternatives like flaxseed. We then offered a useful illustration of the natural characteristics of galliform birds and *Linum*, proposing a symbiosis between the two. The remainder of our review focused directly on flaxseed’s utility as an immune enhancer for poultry. We engaged this by reviewing the following: (i) H/L in flaxseed-fed laying hens, (ii) prospective studies of *S.* Enteritidis, *E. tenella*, and *E. maxima* in flaxseed-fed chickens, and (iii) flaxseed’s ability to improve immune function by antagonizing vitamin B6 in laying hens. Multiple studies indicate that flaxseed attenuates H/L, which has important implications for the bird’s resilience against (at least) Gram-negative bacterial infections (e.g., *S. enterica*). Studies on H/L also bring forth questions about the role of SCFAs as modifiers of adaptive immune capacity, in particular as HDACis. There is a tremendous gap in the understanding of how HDACis regulate poultry immunity, given that all extant knowledge is mammalian centric. Prospective microbiological work indicates that flaxseed accelerates chicken recovery from infection with *S.* Enteritidis and *E. tenella*, which corroborates findings that flaxseed attenuates H/L in poultry. Moreover, our review of published work suggests that 10% defatted flaxseed could be a superweapon against *Eimeria*, and perhaps against microbes at large. However, work is needed to understand why *E. maxima* is resilient when chickens are provided whole flaxseed. This review also illustrated the importance of vitamin B6 antagonism in medicinal plants, and we detailed the mechanism of action by which flaxseed (or specifically, 1ADP) antagonizes vitamin B6 in poultry. It seems likely that vitamin B6 antagonism offers unique benefits that separate whole flaxseed from defatted flaxseed. For example, 15% whole flaxseed’s acceleration of PEMT should help to protect the immune-promoting role of VLDL and HDL. In contrast, 10% defatted flaxseed’s metformin-like activity should increase immunity by safeguarding the chicken’s reproductive physiology and by increasing thymidine bioavailability. In summary, this review merges numerous sources of information into a succinct framework that can be used to better understand flaxseed’s immunological role in the chicken, and thereby promote novel research questions for poultry science.

## Abbreviations

1ADP	1-amino D-proline
5DR	5′-deoxyribonuclease
*A. galli*	*Ascaridia galli*
AGP	Antibiotic growth promoter
AMP	Adenosine monophosphate
AMPK	AMP-activated protein kinase
ATP	Adenosine triphosphate
Breg	Regulatory B-cell
dTMP	Deoxythymidine monophosphate
dTTP	Deoxythymidine triphosphate
dUMP	Deoxyuracil monophosphate
DNA	Deoxyribonucleic acid
ERE	Estrogen response element
*E. maxima*	*Eimeria maxima*
*E. tenella*	*Eimeria tenella*
EU	European Union
FAO	Fatty acid oxidation
*G. biloba*	*Gingko biloba*
HDACi (or HDACis)	Histone deacetylase inhibitor (or histone deacetylase inhibitors)
HDL	High-density lipoprotein
H/L	Heterophil to lymphocyte ratio
IFN-γ	Interferon gamma
IL (e.g., IL-1β)	Interleukin
*L. utassitissimum*	*Linum utassitissimum* (common flax)
LPS	Lypopolysaccharide
Ma	Million years ago
MPN	4′-methylpyridoxine or 4′-methoxypyridoxine
MTHFR	Methylenetetrahydrofolate reductase
PC	Phosphatidylcholine
PE	Phosphatidylethanolamine
PEMT	Phosphatidylethanolamine methyltransferase
p.i.	Post-inoculation
PLP	Pyridoxal 5′-phosphate
*S. enterica*	*Salmonella enterica*
*S*. Enteritidis	*Salmonella enterica* Enteritidis serovar
*S*. Typhimurium	*Salmonella enterica* Typhimurium serovar
SAH	S-adenosylhomocysteine
SAM	S-adenosylmethionine
SCFA	Short-chain fatty acid
SHMT1	Serine hydroxymethyltransferase (cytosolic)
SHMT2	Serine hydroxymethyltransferase (mitochondrial)
TNF-α	Tumor necrosis factor-α
TLR (e.g., TLR4)	Toll-like receptor
US	United States
USDA	United States Department of Agriculture
VLDL	Very-low-density lipoprotein
